# Some Impressions of a New Medical School in Africa

**Published:** 1970-10

**Authors:** J. M. Naish

**Affiliations:** Consultant Physician, Frenchay Hospital, Bristol


### Some Impressions of a New Medical School in Africa

**J. M. Naish, M.D., F.R.C.P.**
Consultant Physician, Frenchay Hospital, Bristol

I travelled to Lagos early in October 1968 to head the Department of Medicine in the Medical College of the University for one academic year. My first misgivings about the organisation I was to join came from the extremely vague and unbusinesslike way in which I was called to this task, and from the obvious sense of muddle which surrounded my appointment both before and after arrival. However, travel arrangements went smoothly and I was soon driven to a vast house on the edge of a swamp, although I had specifically stated that I wanted a small flat in the hospital compound. The mistake was recitified within twenty-four hours and I was installed in the guest flat looking out over a green lawn dotted with palms and flowering trees, not far from the Nurses' Training School and within five minutes walk of the hospital.

My next surprise was to find that half the senior members of my Department were away; that a high proportion of the Professors of other Departments were away, and that my predecessor had left two weeks previously without leaving me any form of note, letter or confidential document about the running of the Department. All was to be revealed in due course, and I was going to have to work for it. No one volunteered any of the information which would have been very helpful to an incoming head. I was later to learn that this "clammishness" is a Yoruba characteristic, and that since Lagos culture is predominantly Yoruba, the treatment given to me did not imply any basic hostility. Nevertheless, it cannot fail to be disturbing to a newcomer, and it may denote a lack of sympathy and psychological insight.

In the ensuing months I was to learn that absences of academic staff, particularly professorial staff, were of even greater extent and duration than those of wholetime academic staff in Britain. One or the reasons for this is that the African loves travelling, especially to Europe and America. Many of them are appointed as special W.H.O. advisers on this and that subject. Because the ground rules of the W.H.O. demand that a certain proportion of their special advisers or members of expert committees should be from Africa, and since the number of possible experts in Africa is small, each individual may find that he gets one or two invitations a year to visit Geneva or the Brazzaville Headquarters of W.H.O. Then there are the international conferences at which papers on "The incidence of X, Y or Z disease in West (Central, East, North) Africa" are eagerly accepted, and where expenses are sometimes paid by the organisers or by pharmaceutical companies. The frequent absences of senior staff in an institution whose staff is not large cause some of the difficulties which one soon meets in the running of a hospital and of the postgraduate educational programme. Junior consultants left as acting heads of the Departments are often unwilling to take decisions in the absence of their chief, and problems tend to be swept under the carpet.

The hospital in Lagos is administered separately from the medical school which is a "college" of the University with a Provost as its academic head. Administration tends, therefore, to be the province of doctors rather than of professionals. In England most of us have, I think, decided that things work better if you leave administration to professional administrators and rely on doctors to give expert advice through special committees. My experience in Nigeria did nothing to alter this view. In fact, I was distressed by the administrative confusion, little of which could be blamed on a particular individual, but which was essentially due to the wrong administrative framework and the lack of a sufficiently skilled and powerful lay administrator.

Administration comes into every aspect of life in a hospital, even if in such simple matters as arrangements to see that X-rays do not get lost and that blood specimens reach the laboratory. Such elementary services do not always work properly in Lagos. The frustrations caused by the creakings of the system, which certainly did not lack clerks, messengers, porters and ward maids, to operate it, are the first concern of the consultant who tries to encourage a decent standard of work by his house officers. The average H.P. has sixteen beds to look after, one admission day a week and one admission weekend every five weeks. Some of the cases admitted are seriously ill patients with tetanus, typhoid, tuberculous meningitis, pyaemia, severe anaemia, sickle cell crises, pneumonia, heart failure and cerebral vascular accidents. The H.P. has to see the patients in Casualty and to "find a bed". This often baffles him, as he is unable to exercise enough determination to discharge a patient who is nearly well to make room for the new admission and he fears trouble from the seniors more than he worries about the length of time the patient stays in Casualty. Such patients may lie all night in Casualty sometimes receiving quite complicated treatment such an intravenous therapy, because nobody has the determination to send a convalescent patient out. This is not too serious, but the practice which had to be vigorously opposed was that of leaving the patient in Casualty without a decision as to who was responsible for him or of sending the patient away and telling him to report to the General Hospital which is four and a half miles away and almost certainly also not able to admit him.

If the H.P. tries to get backing from a registrar or a consultant in order to discharge a patient so as to admit another one, he runs up against several possible difficulties. Firstly, senior staff may be absent or because of the appalling telephone system unobtainable. Secondly, they may countermand his actions and tell him to send the patient away to another hospital. It is scarcely surprising that many interns become disillusioned and cynical, and that they soon come to take the line of least resistance. If they do "take on" the patient either by treating him in Casualty or by admitting him, they are then up against difficulties in obtaining the results of simple investigations such as X-rays and blood examinations. Much time must be spent in chasing films and in visits to the laboratory. Many H.P.s. will not take this trouble and simply report next morning that "the X-ray has been lost" or "the blood sugar result is not available". Of course, if the consultant himself takes the trouble, he will get the information he needs, but it involves walking to the several departments and tedious delays during which he comes across a great deal of muddle and bureaucracy. Forms have to be signed for nearly every need, and one is lucky if the forms aren't in triplicate. My experience was that I could nearly always get the information I needed, but at the expense of time and the discomforts of walking around the hospital in a temperature of 85° and humidity of 90 per cent. The H.P. if he were to take such trouble, and some of them don't even try, would find greater difficulties than I experienced. The "bigger" the man, the easier it is to over-ride the "wilful obstructionism" which is such a feature of lower-rank administration in Yoruba-land.

The cynical and disillusioned H.P. who has lost faith in his seniors and in the workings of the hospital is however happy in his personal life. He has a small flat in the hospital compound, and almost certainly owns a big car for which he can sometimes obtain a loan from the hospital authorities. He is not often seen on the wards in the afternoon, and is very difficult to trace by telephone.

Personally, I had the best H.Ps. to work with, they showed plenty of loyalty, and they tried "hard" judged by their own standards. Nevertheless, it must be said that the house doctor in Lagos has a work output and competence well below that of his opposite number in Bristol.

This is strange, because as students they have extremely good clinical experience. The wards are full of patients with ascites, enlarged organs, failing hearts and interesting anaemias. If keen, a student has plenty of opportunity to do lumbar punctures, tap abdomens and open abscesses, and most of them are keen. Unfortunately, they are keener on absorbing theoretical knowledge than they are on doing things, and this is a trend that one meets all along the line in the Nigerian medical scene.

The students turn up regularly on ward rounds and at clinics. If they are late it is because it is the standard practice in Lagos to be late for everything. I found them interested, but with an even greater penchant (if that is possible) than the English students for wanting to absorb details of rare diseases they are never likely to see. Asked a question they tend to remain dumb as if they don't know the answer, or alternatively to wrap the answer up in such a swaddling cloth of verbiage that the interlocutor is confused. The Professor of Anatomy reported to me that one student, faced with a question at an oral examination remained silent for 5.51 minutes. My efforts were directed towards obtaining reasonably prompt and unambiguous answers from senior students, and by a combination of mild scorn and underlying humour did manage to make some impression on their ingrained habit of "clammishness". It is normally regarded as a severe blow to prestige to answer "I don't know", or to give a short answer which happens to be wrong. The whole cultural background and upbringing encourages them to be vague and evasive in the face of questions for which they cannot find the text-book answer. This tendency is, of course, very bad for the development of scientific habits of thought, and it hinders them from facing real situations with crispness. The job of the medical teacher is to break through this cultural crust, and to get them to think on their feet. That it can be done is proved by the fact that some Nigerians who have spent some years of their life in Europe or America are very capable of quick and flexible thinking. I also noticed that with practice the capacity of some students to think clearly and respond promptly did improve.

Much of what I have written must sound very depressing to an English reader, but one has to realise that African medical schools are very new, and that to have started three schools in Nigeria and to be producing about a hundred doctors a year is a considerable achievement. Nursing standards are, I think, fairly good, though procedures take rather a long time. The one point on which the Lagos Teaching Hospital was superior to any hospital I have worked in except Southmead was in the standard of catering. The catering officer, a Nigerian trained in the north of England, does a first class job, the patient's food is good and plentiful and the staff grumble very little. This catering officer has all the qualities which the Nigerians need if they are to overcome their "under development"; he is brisk, efficient, punctual, has a good memory and makes crisp decisions easily.

I must try to paint a picture of the scene outside the hospital. Lagos has almost as many doctors per head of population as has Bristol, and is therefore completely atypical. The capital of one of the Northern States with a population of over 100,000 has just one elderly doctor. In Lagos, private practice thrives and there are one or two expatriate partnerships which provide their owners with a very good income. Office calls are said to cost £3 and home calls £6-£10. Nigerian doctors in government service stop work at 2.0 p.m. and after that they can look after their private practices. It is widely believed in Lagos that teachers in the College of Medicine are doing private practice, though it is strictly forbidden. Some are undoubtedly paid "retainers" by large firms.

The standards of medicine are often poor. There is a great deal of polypharmacy and blunderbuss treatment of fevers with mixed antibiotics. There is an urban neurosis about hypertension, for which I think the doctors themselves are responsible, and there must be hundreds of obese patients who are swallowing two or three different types of hypotensive drugs every day of their lives. Patients with hypertension even if uncomplicated are kept off work for years sometimes, and patients with innocent cardiac murmurs are not infrequently turned into chronic invalids. Vitamins are prescribed in large quantities, though I saw very little clinical evidence of vitamin deficiencies. Facilities for the treatment of tuberculosis are woefully inadequate so that open cases have to travel daily by bus to get their Streptomycin injections, and it is extremely difficult for the new patient to be accepted for treatment. The service just seems to be overwhelmed by numbers and no doubt also suffers from lack of money. The problem is typical of a developing country and indicates some failure of central medical planning, for too much money is being spent on electronic equipment which rarely works for long, and on "prestige" projects, and not enough on the basic preventive and curative services. The high death rate from tetanus alone suggests that much more should be done to publicise and provide prophylaxis. Much blindness could be prevented if hygiene was better and if treatment for onchocerciasis was sought earlier. Unfortunately, the first port of call for the sick poor is a native doctor: he is cheaper and he responds to their own ideas about sickness and health. Though often shrewd, these men inevitably prolong the period of waiting between first symptoms and definitive diagnosis. This is particularly important in cases of tuberculosis, meningitis and typhoid, and in many childhood diseases. One learns to disbelieve the first history obtained from the patient or relatives, as they are afraid to admit that the illness has gone on for three weeks and will only confess to one or two days. If believed, such statements can lead the clinician towards a wrong diagnosis. In the end the safest course is a veterinary approach followed by a history-taking of the police detective type.

The expatriate and more especially the expatriate's wife needs a car and some strong interests to survive the physical and emotional climate. If one likes "high life" and "soul" then Nigeria must be fascinating. By day, tennis, badminton and golf can all be played after an adjustment to the climate has been made. I found the Yacht Club a friendly place where competitive dinghy sailors and easy-going curry-lunchers mixed in various states of undress on a green lawn at the edge of the harbour. If a breeze was blowing it blew there, and when sailing in the harbour dressed in a pair of bathing trunks one rarely felt the heat, though the vertical sun's rays could burn the brownest back. There are numerous creeks radiating out from Lagos Harbour up which one can sail or speed-boat for more than twenty miles, and there are many places of access to the interminable sandy beaches facing the South Atlantic. A picnic has to be well organised with food and cold drinks packed in thermostatic boxes, and a destination where there is a hut or deep enough shade to eat in comfort. Water ski-ing conditions were ideal for the richer Europeans and Africans, and swimming in formal pools is becoming more popular, especially with the younger Africans. There is also excellent game fishing in the harbour during the winter months. The coast around Lagos could become an important tourist attraction when developed to the extent of parts of the East African coast, but in its present state it provides the essential "lung" for the city dweller wanting to escape from the heat, congestion, noise and road perils of the overcrowded urban areas.

The expatriate in Lagos who doesn't care for water-sports should, if possible, have some strong hobby or interest, even if it is only bridge in the evenings. The study of native art and archaeology, bird-watching, up-country shooting and camping expeditions can all lighten the gloom of frustration which besets most Westerners in an environment where the norms of efficiency are so different from his own.

Finally I would like to say that I found my experience in Lagos to be challenging and full of interest, though it was too frustrating to be really pleasant or comfortable. One needs toughness to withstand the daily knocks and, of course, the cultural isolation. Expatriate doctors need either to be very young, or to have power to institute change within their field of work. I would not advise a senior registrar to take a post as a lecturer unless he has a definite programme of research and the means to carry it through independent of the institution in which he works. If he has to rely on "normal channels" for supply of equipment, facilities or backing from above he is likely to meet many obstacles. Some Nigerian doctors are very expert and very pleasant characters; one feels really sorry for them having to work in a system which is just as frustrating for them as it is for an expatriate. They need all the encouragement and help they can get, and I count myself lucky to have had their friendship while in Lagos.

I very much hope that in my attempts to be truthful and realistic about the situation, I have not written anything that would deter the really adventurous and motivated doctor or nurse from going our there to research, to teach, to learn and to help.

We are an insular race living a comfortable life in a smooth running country. From abroad life looks pretty good in England, but it's a very different life from that which the vast majority of the world live. I would always hope that more of our young will go out and share that life and experience some of their difficulties.

